# Supplementary material for: Effectiveness and Safety of Glucosamine in Osteoarthritis: A Systematic Review
Source: Pharmacy (Basel). 2023 Jul 14;11(4):117. doi: 10.3390/pharmacy11040117 (PMC10366893; doi:10.3390/pharmacy11040117)
Supplement: Supplementary file 1 [file pharmacy-11-00117-s001.zip › pharmacy-2446800-supplementary.pdf]

pharmacy

MDPI

Supplementary Materials: Effectiveness and Safety of Glucosamine in Osteoarthritis: A Systematic Review

Nam Xuan Vo, Ngan Nguyen Hoang Le, Trinh Dang Phuong Chu, Huong Lai Pham, Khang Xuan An Dinh, Uyen Thi Thuc Che, Thanh Thi Thanh Ngo, Tien Thuy Bui

3.2. Risk of bias assessment

Table S1. Risk of bias following Cochrane criteria.

No.	References	Low / High
1	Rindone et al., 2000 [1]	Low
2	Regisnter et al., 2001 [2]	Low
3	Hughes et al., 2002 [3]	Low
4	Pavelka et al., 2002 [4]	Low
5	Cibere et al., 2004 [5]	Low
6	McAlindon et al., 2004 [6]	Low
7	Clegg et al., 2006 [7]	Low
8	Herrero-Beaumont et al., 2007 [8]	Low
9	Frestedt et al., 2008 [9]	Low
10	Giodarno et al., 2009 [10]	Low
11	Petersen et al., 2011 [11]	Low
12	Madhu et al., 2013 [12]	Low
13	Nieman et al., 2013 [13]	Low
14	Kwoh et al., 2014 [14]	Low
15	Fransen et al., 2015 [15]	Low

3.3. Effectiveness of Glucosamine on Knee osteoarthritis

Table S2. The WOMAC score changes of pain in patients of placebo versus treated with glucosamine.

Study and year	Placebo			Glucosamine			Std. Mean Difference	
	Total	Mean	SD	Total	Mean	SD	Weight	IV, Fixed, 95% CI
Fransen et al., 2015	151	-2.1	3.45	152	-22	3.55	18.8%	0.03 [-0.20, 0.25]
Nieman et al., 2013	51	-0.9	2.86	49	-2.4	2.8	6.0%	-0.53 [-0.92, -0.13]
Frestedt et al., 2008	16	-2.9	22.16	19	-12.3	16.26	2.1%	-0.48 [-1.15, 0.02]
Herreo-Beaumont et al., 2007	104	-1.8	4.16	106	-2.7	3.15	12.9%	-0.24 [-0.51, 0.03]
Clegg et al., 2006	313	-86.1	114.2	317	-82.9	115.4	39.1%	0.03 [-0.13, 0.18]
Cibere et al., 2004	66	-28	104	71	-25	98	8.5%	0.03 [-0.31, 0.36]

McAlindon et al., 2004	104	-2.5	3.8	101	-2	3.4	12.7%	0.14 [-0.14, 0.41]
**Total**	**805**			**815**			**100.0%**	**-0.04 [-0.13, 0.06]**

Table S3. The WOMAC score changes physical function in patients of placebo versus treated with glucosamine.

Study and year	Placebo			Glucosamine			Std. Mean Difference	
	Total	Mean	SD	Total	Mean	SD	Weight	IV, Fixed, 95% CI
Fransen et al., 2015	151	-3.9	12.85	152	-3.9	12.68	19.1%	0.00 [-0.23, 0.23]
Nieman et al., 2013	36	-3	9.34	36	-8.2	9.34	4.4%	-0.55 [-1.02, -0.08]
Frestedt et al., 2008	16	-7	23.55	19	-10.6	16.31	2.2%	-0.18 [-0.84, 0.49]
Herreo-Beaumont et al., 2007	104	-5.5	11.45	106	-9.2	10.51	13.1%	-0.34 [-0.61, -0.06]
Clegg et al., 2006	313	-227.4	362.7	317	-222.3	388.3	39.7%	0.01 [-0.14, 0.17]
Cibere et al., 2004	66	-63	318	71	-58	270	8.6%	0.02 [-0.32, 0.35]
McAlindon et al., 2004	104	-4.6	9.6	101	-5.2	9.5	12.9%	-0.06 [-0.34, 0.21]
**Total**	**790**			**802**			**100.0%**	**-0.07 [-0.17, 0.03]**

Table S4. The WOMAC score changes of pain stiffness in patients of placebo versus treated with glucosamine.

Study and year	Placebo			Glucosamine			Std. Mean Difference	
	Total	Mean	SD	Total	Mean	SD	Weight	IV, Fixed, 95% CI
Giordano et al., 2009	30	0	0.3	30	-1	0.3	20.4%	-1.00 [-1.15, -0.85]
Frestedt et al., 2008	9	-0.5	1.5	14	-0.8	1.9	8.6%	-0.30 [-1.70, 1.10]
Clegg et al., 2006	313	-1.5	2.1	317	-1.4	2.1	19.3%	0.10 [-0.23, 0.43]
McAlindon et al., 2004	104	0.8	1.5	101	0.7	1.6	18.4%	-0.10 [-0.52, 0.32]
Pavelka et al., 2002	55	0.1	0.7	66	-0.3	1.5	18.5%	-0.40 [-0.81, 0.01]
Regisnter et al., 2001	71	0	2.2	68	0	2.3	14.8%	0.00 [-0.75, 0.75]
**Total**	**582**			**596**			**100.0 %**	**-0.30 [-0.82, 0.21]**

3.4. Safety

3.4.1. Adverse events

Table S5. The adverse events comparison between placebo and glucosamine (including patients withdrawn caused of adverse events).

Study and year	Relative risk (RR) 95% CI	Weight %
Fransen et al., 2015	7.33 [0.96, 56.00]	0.33
Kwoh et al., 2014	0.39 [0.13, 1.20]	3.53
Rozendaal et al., 2008	2.29 [0.26, 20.13]	0.71
Herrero-Beaumont et al., 2007	0.54 [0.19, 1.57]	6.18
Clegg et al., 2006	0.71 [0.31, 1.60]	8.97
McAlindon et al., 2004	0.50 [0.07, 3.75]	1.83
Pavelka et al., 2002	1.05 [0.46, 2.39]	6.57
Reginster et al., 2001	1.07 [0.70, 1.65]	14.26
**Total (I^2 = 24.3%, p-value = 0.236)**	**0.90 [0.66, 1.23]**	**42.37**

Table S6. Frequency of adverse events reported in patients using Glucosamine Sulphate.

Study and year	Glucosamine sulphate (N)	Adverse events						
		Infections	MU	CNS	GI	CV	Skin	Others
Pavelka et al., 2002 [4]	101	29	30	ND	25	23	10	14
Noack et al., 1994 [16]	126	0	0	2	5	0	1	0
Reginster et al., 2001 [2]	106	ND	ND	31	27	21	ND	4
McAlindon et al., 2004 [6]	101	ND	5	2	4	ND	ND	7
Herrero-Beaumont et al., 2007 [8]	106	12	10	3	11	ND	ND	8
Rozendaal et al., 2008 [17]	111	ND	ND	66	58	21	ND	7
Hughes et al., 2002 [3]	39	1	9	1	0	ND	0	8

* Abbreviations: ND: no data; GI: gastrointestinal; CV: cardiovascular; CNS: central nervous system; MU: musculoskeletal

References

1. Rindone, J.P., et al., *Randomized, controlled trial of glucosamine for treating osteoarthritis of the knee.* West J Med, 2000. **172**: p. 91-4.
2. Reginster, J.Y., et al., *Long-term effects of glucosamine sulphate on osteoarthritis progression: a randomised, placebo-controlled clinical trial.* Lancet, 2001. **357**: p. 251-6.
3. Hughes, R. and A. Carr, *A randomized, double-blind, placebo-controlled trial of glucosamine sulphate as an analgesic in osteoarthritis of the knee.* Rheumatology (Oxford), 2002. **41**: p. 279-84.
4. Pavelká, K., et al., *Glucosamine sulfate use and delay of progression of knee osteoarthritis: a 3-year, randomized, placebo-controlled, double-blind study.* Arch Intern Med, 2002. **162**: p. 2113-23.
5. Cibere, J., et al., *Randomized, double-blind, placebo-controlled glucosamine discontinuation trial in knee osteoarthritis.* Arthritis Rheum, 2004. **51**: p. 738-45.
6. McAlindon, T., et al., *Effectiveness of glucosamine for symptoms of knee osteoarthritis: results from an internet-based randomized double-blind controlled trial.* Am J Med, 2004. **117**: p. 643-9.
7. Clegg, D.O., et al., *Glucosamine, chondroitin sulfate, and the two in combination for painful knee osteoarthritis.* N Engl J Med, 2006. **354**: p. 795-808.
8. Herrero-Beaumont, G., et al., *Glucosamine sulfate in the treatment of knee osteoarthritis symptoms: a randomized, double-blind, placebo-controlled study using acetaminophen as a side comparator.* Arthritis Rheum, 2007. **56**: p. 555-67.

9. Frestedt, J.L., et al., *A natural mineral supplement provides relief from knee osteoarthritis symptoms: a randomized controlled pilot trial.* Nutr J, 2008. **17**: p. 9.
10. Giordano, N., et al., *The efficacy and tolerability of glucosamine sulfate in the treatment of knee osteoarthritis: A randomized, double-blind, placebo-controlled trial.* Curr Ther Res Clin Exp, 2009. **70**: p. 185-96.
11. Petersen, S.G., et al., *Nonsteroidal anti-inflammatory drug or glucosamine reduced pain and improved muscle strength with resistance training in a randomized controlled trial of knee osteoarthritis patients.* Arch Phys Med Rehabil, 2011. **92**: p. 1185-93.
12. Madhu, K. and M.J.S. K Chanda, *Safety and efficacy of Curcuma longa extract in the treatment of painful knee osteoarthritis: a randomized placebo-controlled trial.* Inflammopharmacology, 2013. **21**: p. 129-36.
13. Nieman, D.C., et al., *A commercialized dietary supplement alleviates joint pain in community adults: a double-blind, placebo-controlled community trial.* Nutr J, 2013. **12**: p. 154.
14. Kwoh, C.K., et al., *Effect of oral glucosamine on joint structure in individuals with chronic knee pain: a randomized, placebo-controlled clinical trial.* Arthritis Rheumatol, 2014. **66**: p. 930-9.
15. Fransen, M., et al., *Glucosamine and chondroitin for knee osteoarthritis: a double-blind randomised placebo-controlled clinical trial evaluating single and combination regimens.* Ann Rheum Dis, 2015. **74**: p. 851-8.
16. Noack, W., et al., *Glucosamine sulfate in osteoarthritis of the knee.* Osteoarthritis Cartilage, 1994. **2**: p. 51-9.
17. Rozendaal, R.M., et al., *Effect of glucosamine sulfate on hip osteoarthritis: a randomized trial.* Ann Intern Med, 2008. **148**: p. 268-77.
